# Comparative analysis of clinical and immunological profiles across Omicron BA.5.2 subvariants using next-generation sequencing in a Chinese cohort

**DOI:** 10.3389/fcimb.2023.1288914

**Published:** 2023-10-30

**Authors:** Jianliang Huang, Mingkai Xia, Rangjiao Liu, Shaobo Wang, Xinyi Duan, Jiong Peng, Enping Li, Yanping Zhou, Chengyou Li, Quan Zhang, Jixian Tian, Xinjian Wang, Zhongrui Su, Jun Tan, Bo Peng, Jianhui Zhang, Jin Li, Lizhong Dai, Mingsheng Lei

**Affiliations:** ^1^ Zhangjiajie Hospital Affiliated to Hunan Normal University, Zhangjiajie, China; ^2^ Sanway Clinical Laboratory, Changsha, China; ^3^ Sansure Biotech Incorporation, Changsha, China; ^4^ Zhangjiajie College, Zhangjiajie, China

**Keywords:** Omicron subvariants, clinical characteristic, immune function, disease severity, mutation sites, NGS

## Abstract

**Objective:**

The Omicron BA.5.2 variant of SARS-CoV-2 has undergone several evolutionary adaptations, leading to multiple subvariants. Rapid and accurate characterization of these subvariants is essential for effective treatment, particularly in critically ill patients. This study leverages Next-Generation Sequencing (NGS) to elucidate the clinical and immunological features across different Omicron BA.5.2 subvariants.

**Methods:**

We enrolled 28 patients infected with the Omicron variant, hospitalized in Zhangjiajie People’s Hospital, Hunan, China, between January 20, 2023, and March 31, 2023. Throat swabs were collected upon admission for NGS-based identification of Omicron subvariants. Clinical data, including qSOFA scores and key laboratory tests, were collated. A detailed analysis of lymphocyte subsets was conducted to ascertain the extent of immune cell damage and disease severity.

**Results:**

Patients were infected with various Omicron subvariants, including BA.5.2.48, BA.5.2.49, BA.5.2.6, BF.7.14, DY.1, DY.2, DY.3, and DY.4. Despite having 43 identical mutation sites, each subvariant exhibited unique marker mutations. Critically ill patients demonstrated significant depletion in total lymphocyte count, T cells, CD4, CD8, B cells, and NK cells (*P < 0.05*). However, there were no significant differences in clinical and immunological markers across the subvariants.

**Conclusion:**

This study reveals that critically ill patients infected with different Omicron BA.5.2 subvariants experience similar levels of cellular immune dysfunction and inflammatory response. Four mutations - ORF1a:K3353R, ORF1a:L3667F, ORF1b:S997P, S:T883I showed correlation with immunological responses although this conclusion suffers from the small sample size. Our findings underscore the utility of NGS in the comprehensive assessment of infectious diseases, contributing to more effective clinical decision-making.

## 1 Introduction

Since the discovery of the ‘coronavirus disease 2019’ (COVID-19) towards the end of 2019, the novel coronavirus (SARS-CoV-2) has continued to evolve and has given rise to mutant strains such as Alpha, Beta, Gamma, Delta, Lambda, and Omicron. Currently the Omicron strain is predominant all over the world. Compared to the previous strains such as Alpha and Delta, Omicron appears to cause less severe clinical symptoms and is more likely to infect the upper respiratory tract. As a result, the proportion of severe cases, hospitalizations, and mortality rates have decreased (Sievers et al., 2022). However, the Omicron strain’s infectivity and immune evasion continue to increase as a considerable number of mutations in the S protein (Polatoğlu et al., 2023). On November 26, 2021, the WHO classified it as a variant of concern (VOC).

The genome sequences of the SARS-CoV-2 detected in China between September 26, 2022 and April 13, 2023 were all Omicron variants, covering 106 evolutionary branches, with the most prevalent strains being BF.7.14, DY.2, DY.4, BA.5.2.48, and DY.1, DY.3 (Chinese Center for Disease Control and Prevention). BA.5.2 was caused by the mutation BA.5+ORF9b:D16G;BA.5.2.48, BF.7.14, and DY variants were derived from BA.5.2, containing novel mutation sites S:A57S, ORF1a:T1788M, and S:C124F. Virus mutations often result in modifications to the disease manifestation, symptoms, and defining characteristics. Previously it was assumed that BA.5.2.48 strain and BF.7 strain might be different in their ability to cause disease, however, A comprehensive search was conducted on Web of Science, PubMed, and China National Knowledge Infrastructure to identify relevant literature on Omicron BA.5.2 subvariants. limited studies were found on the clinical characteristics of BA.5.2.48, BA.5.2.49, and BF.7.14. until now no report is available to describe the clinical features of the DY subvariant.

T lymphocytes were discovered to be crucial in the process of the novel corona virus’s resistance (Toor et al., 2020; Tirelli et al., 2023). Despite a significant number of mutations in the virus, the Omicron strain is still attacked by the T cell immune response. However, patients with severe COVID-19 frequently have a cytokine storm, which results in excessive T cell activation and exhaustion, a large number of T cells being destroyed, and patients could die from immune function failure. It is currently unclear, nevertheless, if distinct Omicron variants (BA.5.2 evolutionary strains) could produce different harm to human immune function. In this study, the nucleic acids were extracted from the throat swabs of all enrolled patients and sequenced to determine the type of variation. General patient data and laboratory examination (including lymphocyte subsets, etc.) data were collected to investigate the relationship between subvariants of Omicron BA.5.2.and clinical characteristics, immune function, and disease severity.

## 2 Materials and methods

### 2.1 Study participants

The present study was an observational study of patients with SARS-CoV-2 infection were admitted to Zhangjiajie People’s Hospital (Hunan, China) between January 20, 2023, and March 31, 2023 were included in this study. All patients met the diagnosis criteria of SARS-CoV-2 infection, as outlined in the National Health Commission of China’s Trial Ninth Edition (National Health Commission State Administration of Traditional Chinese Medicine, 2022). Patients were excluded if they had following conditions:1).Patients with severe immunodeficiency such as HIV, end-stage tumors, and long-term use of immunosuppressants; 2).The quality of samples did not meet the detection standards (such as nucleic acid PCR CT value >32, etc.); 3).Patients who cannot cooperate to complete the sample collection; 4).Patients who did not have sufficient clinical data; 5).Patients who refuse to participate in this study. A total of 28 people were included in this study (**Supplementary Figure 1**), including 10 patients with severe COVID-19 (clinically diagnosed as severe or critical) and 18 non-severe patients (clinically diagnosed as mild or moderate). (**Supplementary Table 1**). This study was approved by the Medical Ethics Review Committee of Zhangjiajie People’s Hospital (IRB-2022237), and all patients signed informed consent.

### 2.2 Data and sample collection

Throat swabs were collected from enrolled patients to detect and identify the Omicron subvariants. The collected samples were stored in a -20°C refrigerator with RNA preservation solution for a short period of time to maintain the integrity of nucleic acids and prevent degradation. Upon admission, the patient’s general health was thoroughly reviewed, other information of underlying diseases, potential complications, and clinical symptoms was gathered. Additionally, the qSOFA score and laboratory tests, including lymphocyte subsets, were assessed to reflect the patient’s immune function. The qSOFA score is a tool used to evaluate the risk of sepsis in patients. The score is calculated based on three criteria: systolic blood pressure of 100 mmHg or lower, shortness of breath of 22 breaths per minute or more, and mental status changes. Each criterion is worth one point, and a score of 2 or higher indicates a high likelihood of sepsis and requires close monitoring (Song et al., 2018).

### 2.3 Flow cytometric analysis

Peripheral blood lymphocytes were detected using a flow cytometer called BriCyte E6, which was purchased from China Mindray Biomedical Electronics Co., Ltd. The flow cytometer collected parameter information such as forward scattered light (FSC), side scattered light (SSC), and fluorescence signals of cells. It then analyzed the subpopulations of T lymphocytes (CD3^+^), the auxiliary/inducible classification and counting of T lymphocytes (CD3^+^CD4^+^), the suppressor/cytotoxic T lymphocytes (CD3^+^CD8^+^) subpopulation, the B lymphocytes (CD3^-^CD19^+^) and NK cells (CD3^-^CD16^+^CD56^+^). The reagents used consisted of a four-color reagent for CD3-FITC/CD8-PE/CD45-PerCP/CD4-APC and another four-color reagent for CD3-FITC/CD16 + 56-PE/CD45-PerCP/CD19-APC. All tests were performed strictly following the instructions and standard operating procedures provided with the kit to ensure accurate test results.

### 2.4 Sequencing and mutation analysis of Omicron subvariants

RNAs were extracted from the 300 µL of samples of throat swabs using a QIAamp Viral RNA Mini Kit. The cDNA synthesis, SARS-CoV-2 sequence enrichment, library amplification, and indexing were performed by using the SARS-CoV-2 Nucleic Acid Diagnosis Kit (Sequencing by Reversible Termination, Sansure Biotech, China). An elution volume of 20-50 μL for each RNA sample was used for cDNA synthesis. After the cDNA reaction, A multiplex PCR reaction was done to amplify the entire SARS-CoV-2 genome using specifically designed primers. The product DNA was diluted to approximately 0.5 ng/μL and 1 ng of DNA was added to the reaction system for DNA fragmentation and library construction. After the reaction was completed, it was purified using 80% fresh ethanol and DNA Clean Beads. The quality of the library was analyzed using the LabChip GX Touch™ DNA 1K Chip^®^. Next-generation sequencing was done by using SE150 of GeneMind’s sequencer (SansureSeq1000, China), at least 8M reads were obtained for each sample. All the sequencing data (FastQ files) of samples can be downloaded from cncb.ac.cn with accession number PRJCA019496.

Low quality and adapter sequences were removed using trim_galore, more than 99% of the remaining raw reads (so-called clean reads) were used for analysis. More than 98% of the clean reads could be mapped to the SARS-CoV-2 reference genome Wuhan-Hu-1(NC_045512.2) using Burrows-Wheeler Alignment tool (BWA-MEM v0.7.17). Subsequently, the mapped reads were assembled into a consensus sequence using samtools (v1.9) and ivar (v1.2.1) tools, and the sites whose depth is lower than the threshold parameter (20x) will be output as N. For phylogenetic analysis, full-length SARS-CoV-2 reference sequences were selected according to the categories of novel coronavirus labels in GISAID database, in which 2 reference sequences were selected respectively from 10 categories: Alpha, Beta, Gamma, Delta, Epsilon, Zeta, Eta, Theta, Iota and Kappa, and the patient’s consensus sequences were classified into lineages using PANGOLIN and into clades using NextClade (v0.10.0). Multi-sequence alignment was performed with MAFFT (v7.271), FastTee was used to construct phylogenetic tree. The mutation frequency of the site was calculated by VarScan (v2.4.4).

### 2.5 Statistical analysis

Measurement data were described by median (interquartile range), Count data were expressed as frequencies with percentages. Comparative analysis between groups was conducted using Kruskall, Mann-Whitney non-parametric rank sum test, Fisher chi-square test and Wilcoxon test. All statistical analyses were performed using SPSS 22.0, and a *P* value less than 0.05 was considered statistically significant.

## 3 Results

### 3.1 Clinical characteristics of patients

This study included a total of 28 Omicron-infected patients; there were 17 males (60.7%) and 11 females (39.3%); 10 patients (35.7%) were classified as severe, and median qSOFA score was 1.0 (0,1.0); median age was 59.0 (50.0, 76.0), Fifty percent of the patients were over 60 years old; Twenty four patients (85.7%) had underlying diseases, mainly hypertension (42.9%), diabetes (17.9%), and coronary heart disease (21.4%); The most prevalent clinical manifestations were fever (42.9%), cough and expectoration (92.9%), shortness of breath (50.0%), sore throat (28.6%), and headache (28.6%); some patients also experienced chills, anorexia, fatigue, diarrhea, muscle ache, and other symptoms. Fifteen patients (53.6%) had complications by the time they were discharged. Common complications included respiratory failure (35.7%), acute respiratory distress syndrome (ARDS) (10.7%), renal insufficiency (10.7%), hepatic insufficiency (14.3%), heart failure (14.3%), hypoalbuminemia (25.0%), acidosis (14.3%), sepsis (17.9%), multiple organ dysfunction syndrome (MODS) (17.9%), and others (**Table 1**).

**Table 1:** General information and clinical characteristics.

Characteristics	Total(n = 28)	BA.5.2.48+BA.5.2.49+BA.5.2.6(n=5)	BF.7.14(n=8)	DY(n=15)	*P* value
Age (years)	59.0 (50.0, 76.0)	65.0 (60.0, 75.0)	55.0 (52.0, 65.0)	57.0 (49.0, 80.0)	0.482
<45	3 (10.7%)	0	0	3(20.0%)	
45-59	11 (39.3%)	1(20.0%)	5(62.5%)	5(33.3%)	
≥60	14 (50.0%)	4(80.0%)	3(37.5%)	7(46.7%)	
Sex					0.522
Male	17 (60.7%)	2(40.0%)	5(62.5%)	10(66.7%)	
Female	11 (39.3%)	3(60.0%)	3(37.5%)	5(33.3%)	
Classification					0.373
Non-Severe pneumonia	18 (64.3%)	2(40.0%)	5(62.5%)	11(73.3%)	
Severe pneumonia	10 (35.7%)	3(60.0%)	3(37.5%)	4(26.7%)	
Basic illness					0.260
Yes	24 (85.7%)	5(100%)	8(100%)	11(73.3%)	
No	4 (14.3%)	0	0	4(26.7%)	
Hypertension	12 (42.9%)	2(40.0%)	4(50.0%)	6(40.0%)	0.882
Diabetes	5 (17.9%)	1(20.0%)	2(25.0%)	2(13.3%)	0.815
Emphysema	3 (10.7%)	1(20.0%)	2(25.0%)	0	0.087
Coronary heart disease	6 (21.4%)	1(20.0%)	3(37.5%)	2(13.3%)	0.488
Chronic renal insufficiency	3 (10.7%)	1(20.0%)	2(25.0%)	0	0.087
Hepatitis	3 (10.7%)	0	2(25.0%)	1(6.7%)	0.261
Tumor	3 (10.7%)	0	1(12.5%)	2(13.3%)	1.000
Clinical symptoms					
Fever	12 (42.9%)	0	4(50.0%)	8(53.5%)	0.121
Chills	5 (17.9%)	0	2(25.0%)	3(20.0%)	0.665
Cough,Expectoration	26 (92.9%)	5(100%)	8(100%)	13(86.7%)	0.683
Hemoptysis	1 (3.6%)	0	1(12.5%)	0	0.464
Shortness of breath	14 (50.0%)	3(60.0%)	6(75.0%)	5(33.3%)	0.164
Anorexia	5 (17.9%)	1(20.0%)	1(12.5%)	3(20.0%)	1.000
Sore throat	8 (28.6%)	3(60.0%)	0	5(33.3%)	0.052
Runny nose,stuffy nose	5 (17.9%)	1(20.0%)	1(12.5%)	5(33.3%)	1.000
Headache	8 (28.6%)	2(40.0%)	2(25.0%)	3(20.0%)	0.865
Nausea	7 (25.0%)	1(20.0%)	2(25.0%)	4(26.7%)	1.000
Weak	8 (28.6%)	1(20.0%)	2(25.0%)	2(13.3%)	1.000
Chest tightness	4 (14.3%)	0	2(25.0%)	4(26.7%)	0.617
Diarrhea	3 (10.7%)	1(20.0%)	2(25.0%)	0	0.087
Muscle ache	5 (17.9%)	2(40.0%)	0	3(20.0%)	0.210
Complication					0.880
Yes	15 (53.6%)	3(60.0%)	5(62.5%)	7(46.7%)	
No	13 (46.4%)	2(40.0%)	3(37.5%)	8(53.3%)	
Respiratory failure	10 (35.7%)	2(40.0%)	5(62.5%)	3(20.0%)	0.117
ARDS	3 (10.7%)	2(40.0%)	1(12.5%)	0	0.045
Heart failure	4 (14.3%)	1(20.0%)	2(25.0%)	1(6.7%)	0.362
Renal insufficiency	3 (10.7%)	1(20.0%)	0	2(13.3%)	0.560
Liver insufficiency	4 (14.3%)	1(20.05)	0	3(20.0%)	0.474
Shock	2 (7.1%)	1(20.0%)	0	1(6.7%)	0.405
Coagulation abnormalities	2 (7.1%)	1(20.0%)	1(12.5%)	0	0.206
Hypoproteinemia	7 (25.0%)	3(60.0%)	2(25.0%)	2(13.3%)	0.097
Acidosis	4 (14.3%)	1(20.0%)	2(25.0%)	1(6.7%)	0.362
Sepsis	5 (17.9%)	1(20.0%)	2(25.0%)	2(13.3%)	0.815
MODS	5 (17.9%)	3(60.0%)	0	2(13.3%)	0.035

### 3.2 Laboratory findings and lymphocytes subsets detection

The lymphocyte counts were found to decline in patients, and the levels of C-reactive protein, interleukin-6, procalcitonin, D-dimer, and troponin in patients were all significantly higher than the normal ranges. (**Table 2**). Median nucleic acid PCR CT value of Lab is 26.86 (22.23,29.69), and the median value of N is 24.05 (20.05,26.75).

**Table 2:** Laboratory findings and qSOFA score.

Laboratory findings	Total (n = 28)	BA.5.2.48+BA.5.2.49+BA.5.2.6 (n=5)	BF.7.14 (n=8)	DY (n=15)	*P* value	Normal value
Leukocyte (×10⁹/L)	4.83 (4.10, 9.14)	10.12 (5.27, 14.26)	7.80 (6.10, 11.76)	4.24 (3.49, 4.47)	0.002	3.50-9.50
Neutrophils (×10⁹/L)	3.97 (2.77, 8.22)	9.51 (3.98, 12.93)	6.04 (5.28, 10.93)	2.88 (1.85, 3.74)	0.009	1.80-6.30
Lymphocytes (×10⁹/L)	0.81 (0.31, 1.00)	0.53 (0.21, 0.91)	0.52 (0.29, 0.99)	0.93 (0.35, 1.14)	0.410	1.10-3.20
Monocytes (×10⁹/L)	0.45 (0.29, 0.60)	0.40 (0.36, 0.87)	0.58 (0.43, 0.87)	0.35 (0.25, 0.49)	0.118	0.10-0.60
Platelets (×10⁹/L)	151.00 (117.00, 198.00)	150.00 (85.00, 175.50)	177.50 (108.50, 267.50)	151.00 (119.00, 194.00)	0.486	100.00-300.00
Hemoglobin (g/L)	113.00 (88.25, 133.25)	108.00 (80.50, 136.50)	82.50 (67.00, 124.50)	125.00 (95.00, 136.00)	0.123	120.00-160.00
CRP (mg/L)	17.67 (4.76, 90.22)	104.15 (28.46, 157.72)	20.01 (7.69, 114.06)	11.14 (2.78, 38.62)	0.094	0-10.00
PCT (ng/mL)	0.29 (0.07, 1.69)	1.31 (0.23, 3.39)	0.61 (0.22, 1.69)	0.10 (0.06, 1.01)	0.226	0-0.05
ALT (U/L)	23.00 (13.00, 39.00)	22.00 (11.50, 33.00)	11.50 (7.25, 37.75)	28.00 (19.00, 40.00)	0.245	0-50.00
AST (U/L)	30.50 (23.00, 40.00)	29.00 (19.50, 55.50)	26.50 (14.25, 43.75)	33.00 (24.00, 39.00)	0.705	0-40.00
Total bilirubin (μmol/L)	8.10 (6.17, 14.92)	8.80 (6.55, 24.40)	7.15 (6.48, 33.95)	8.80 (4.50, 14.70)	0.573	3.40-17.10
Direct bilirubin (μmol/L)	4.35 (3.12, 8.32)	5.00 (4.05, 18.40)	4.70 (3.30, 7.35)	3.20 (2.50, 8.40)	0.182	0-6.18
Indirect bilirubin (μmol/L)	3.75 (2.20, 5.30)	3.80 (2.50, 6.00)	3.10 (2.05, 3.75)	4.20 (2.00, 5.50)	0.564	0-16.00
Blood urea nitrogen (mmol/L)	6.95 (4.64, 10.50)	8.01 (6.23, 13.70))	7.30 (4.99, 18.16)	5.10 (3.50, 8.45)	0.265	3.10-8.00
Creatinine (mg/dL)	87.50 (67.75, 99.85)	79.60 (68.50, 96.80)	90.50 (52.25, 346.00)	87.00 (64.00, 100.60)	0.940	53.00-104.00
Albumin (g/L)	37.40 (33.07, 42.52)	35.50 (33.60, 44.55)	36.65 (32.63, 41.43)	38.60 (32.70, 42.70)	0.903	40.00-55.00
PT (s)	12.66 (11.65, 14.27)	12.90 (12.00, 13.77)	12.18 (11.36, 14.08)	12.70 (12.00, 14.40)	0.519	10.70-14.00
APTT (s)	30.69 (27.80, 32.62)	28.80 (26.14, 30.14)	31.75 (26.89, 35.89)	31.47 (27.80, 32.40)	0.201	21.00-35.00
DDT (μg/mL)	578.42 (240.17, 2883.00)	1387.00 (547.15, 2840.50)	3469.50 (242.25, 6879.04)	459.50 (211.25, 788.65)	0.125	0-400.00
Troponin (pg/mL)	21.81 (7.35, 46.21)	17.50 (9.71, 143.40)	37.35 (21.30, 74.84)	17.80 (6.52, 26.60)	0.235	0-14.00
CK (U/L)	100.50 (49.90, 215.50)	228.00 (53.35, 554.50)	55.50 (27.00, 138.75)	105.00 (57.00, 211.00)	0.214	50.00-310.00
CK-MB (U/L)	14.35 (9.72, 26.05)	11.80 (8.55, 15.57)	15.55 (7.88, 29.18)	15.50 (10.70, 23.20)	0.927	0-25.00
Myoglobin (pg/mL)	56.75 (33.82, 426.07)	399.30 (39.65, 580.90)	153.40 (36.75, 447.60)	56.50 (31.30, 173.20)	0.325	0-70.00
LDH (U/L)	221.00 (197.00, 321.00)	249.00 (184.75, 313.75)	282.50 (213.00, 453.50)	212.00 (195.00, 292.00)	0.419	120.00-250.00
IL-6 (pg/ml)	20.55 (6.35, 47.15)	19.76 (11.99, 223.06)	29.25 (20.33, 82.92)	10.24 (3.56, 35.42)	0.246	0-5.40
qSOFA score	1(0,1)	1(0, 1)	0(0, 1)	1 (0, 1)	0.418	0

Lymphocyte subsets were used to assess a patient’s immune system. In patients with Omicron infection, total T lymphocyte count, CD4^+^ lymphocyte count, CD8^+^ lymphocyte count, B lymphocyte count, and NK cell count were all below normal values, only the corresponding percentage and CD4^+^/CD8^+^ ratio were essentially within the normal range (**Table 3**).

**Table 3:** Lymphocyte subsets and CT value of the nucleic acid.

Project	Total(n = 28)	BA.5.2.48+BA.5.2.49+BA.5.2.6 (n=5)	BF.7.14 (n=8)	DY (n=15)	*P value*	Normal value
Percentage of total T cells (%)	70.29 (57.54, 77.82)	62.14 (36.54, 73.65)	77.76 (57.29, 85.97)	66.22(58.97, 77.73)	0.205	56.00-86.00
Percentage of total CD4 T cells (%)	34.88 (26.00, 51.20)	29.80 (9.14, 43.77)	52.19 (25.48, 69.84)	33.02(31.04, 40.94)	0.138	33.00-58.00
Percentage of total CD8 T cells (%)	26.40 (18.04, 32.96)	26.00 (12.25, 37.76)	22.99 (17.79, 30.07)	27.36(19.72, 37.26)	0.617	13.00-39.00
Percentage of B cells (%) #	17.98 (2.58, 33.14)	20.53 (14.43, 47.33)	7.41 (2.51, 23.50)	15.45(1.98, 33.29)	0.184	5.00-22.00
Percentage of NK cells (%)#	14.21 (10.43, 18.58)	14.21 (8.04, 18.58)	13.35 (10.39, 20.64)	14.63(10.37, 19.36)	0.996	5.00-26.00
CD4/CD8 ratio (/)	1.39 (0.72, 2.30)	0.71 (0.32, 2.22)	1.69 (0.86, 3.44)	1.39(0.89, 2.29)	0.349	0.71-2.78
Total lymphocyte count (μ/l)	553.00 (450.00, 976.00)	485.00 (218.00, 1014.00)	527.00 (468.00, 672.00)	873.00(449.00, 1018.00)	0.594	1530.00-3700.00
Total T cell count (μ/l)	449.00 (193.00, 696.00)	142.00 (114.00, 702.00)	449.00 (238.00, 551.00)	472.00(265.00, 760.00)	0.320	723.00-2737.00
CD4 T cell count (μ/l)	257.00 (88.00, 380.00)	97.00 (25.00, 301.00)	346.00 (86.00, 363.00)	281.00(97.00, 435.00)	0.351	404.00-1612.00
CD8 T cell count (μ/l)	127.00 (78.00, 284.00)	91.00 (36.00, 365.00)	101.00 (80.00, 204.00)	190.00(100.00, 340.00)	0.298	220.00-1129.00
B cell count (μ/l) #	46.00 (20.00, 83.00)	45.00 (25.00, 216.00)	56.00 (26.00, 85.00)	28.50(10.00, 73.00)	0.420	80.00-616.00
NK cell count (μ/l) #	54.00 (51.00, 78.00)	54.00 (26.00, 90.00)	53.00 (51.00, 77.00)	56.00(43.00, 110.00)	0.747	84.00-724.00
Lab value	26.86 (22.23, 29.69)	21.71 (19.49, 30.15)	27.01 (23.43, 28.65)	27.06(23.73, 30.65)	0.647	>35
N value	24.05 (20.05, 26.75)	22.86 (15.85, 26.75)	23.71 (19.40, 25.40)	24.69(20.29, 27.00)	0.497	>35

#: Due to unforeseen circumstances, 9 patients were missing this data(n=19).

### 3.3 Relationship between disease severity and lymphocyte subsets

We categorized all patients as severe or non-severe Omicron infection. Total 18 of them had non-severe conditions, while 10 had severe conditions. Severe patients had significantly lower total lymphocyte counts 389.00 (231.00, 579.00) μ/l, total T lymphocyte counts 165.00 (121.00, 360.00) μ/l, CD4 Lymphocyte counts 85.00 (43.00, 149.00) μ/l, CD8 lymphocyte counts 82.00 (38.00, 185.00) μ/l, and the NK cell counts 52.00 (20.00, 64.00) μ/l. The levels had significantly different between the two groups *(P<0.05)*. Additionally, severe patients had higher IL-6 levels and qSOFA score than those of patients with no-severe Omicron infection *(P <0.001)*. Severe patients’ nucleic acid PCR CT values were marginally lower than those of non-severely unwell patients at admission, but these differences were not statistically significant.(**Table 4**)

**Table 4:** Lymphocyte subsets, IL-6 and PCR CT value of the nucleic acid.

Project	Non-Severe (n=18)	Severe (n=10)	*P* value
Percentage of total T cells (%)	71.64 (62.90, 79.51)	58.02 (50.21, 73.67)	0.121
Percentage of total CD4 T cells (%)	36.67 (31.91, 55.91)	26.03 (17.20, 45.42)	0.146
Percentage of total CD8 T cells (%)	26.05 (19.28, 30.73)	29.79 (16.06, 35.15)	0.796
Percentage of B cells (%) #	8.54 (2.48, 21.91)	21.06 (13.08, 33.43)	0.133
Percentage of NK cells (%)#	13.16 (10.18, 17.78)	17.20 (7.63, 20.48)	0.720
CD4/CD8 ratio (/)	1.39 (0.95, 2.48)	1.04 (0.39, 2.40)	0.286
Total lymphocyte count (μ/l)	910.00 (507.00, 1034.00)	389.00 (231.00, 579.00)	0.001*
Total T cell count (μ/l)	525.00 (364.00, 812.00)	165.00 (121.00, 360.00)	0.001*
CD4 T cell count (μ/l)	356.00 (218.00, 442.00)	85.00 (43.00, 149.00)	0.001*
CD8 T cell count (μ/l)	160.00 (99.00, 330.00)	82.00 (38.00, 185.00)	0.035*
B cell count (μ/l) #	51.00 (13.00, 87.00)	46.00 (36.00, 66.00)	1.000
NK cell count (μ/l) #	64.00 (54.00, 103.00)	52.00 (20.00, 64.00)	0.050*
Lab value	26.86(23.57,30.12)	25.62(20.50,28.60)	0.332
N value	24.47(22.29,27.26)	22.00(16.61,25.21)	0.099
IL-6(pg/ml)	9.80(3.91,21.97)	88.02(33.19,279.40)	<0.001*
qSOFA score	0(0,1)	1(1,2)	<0.001*

#: Due to unforeseen circumstances, 9 patients of Non-Severe pneumonia were missing this data(n=19).

*=P<0.05.

**Figure 1:**
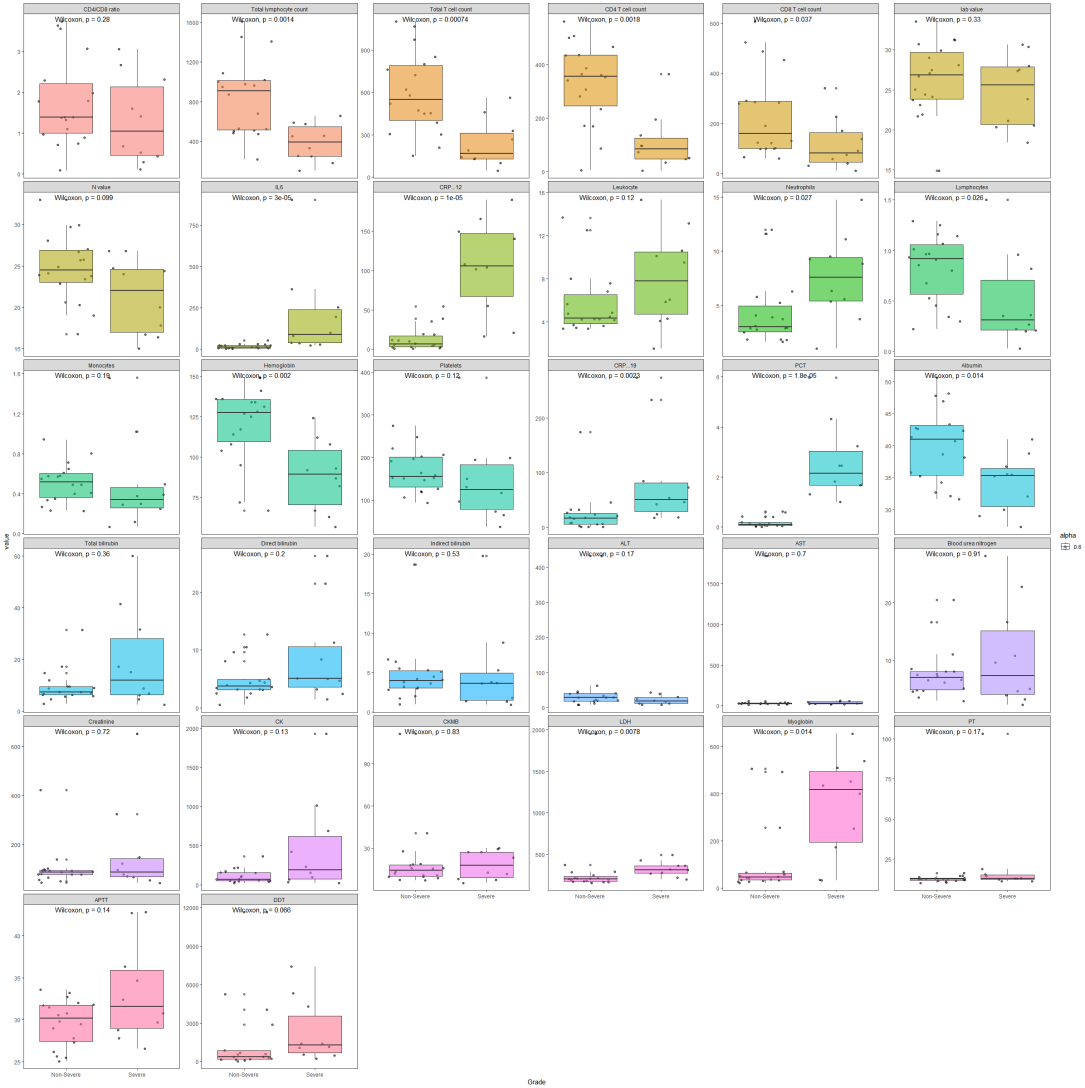
Box plots comparing disease severe and non-severe groups for numerical laboratory testing results.

Box plots comparing the disease severe or non-severe groups for numerical laboratory testing results are illustrated in **Figure 1**. Wilcoxon p values are labeled for each comparison, which are consistent with the p values in **Table 4**.

### 3.4 Mutation sites of Omicron subvariants BA.5.2.48, BA.5.2.49, BA.5.2.6, BF.7.14 and DY

**Figure 2:**
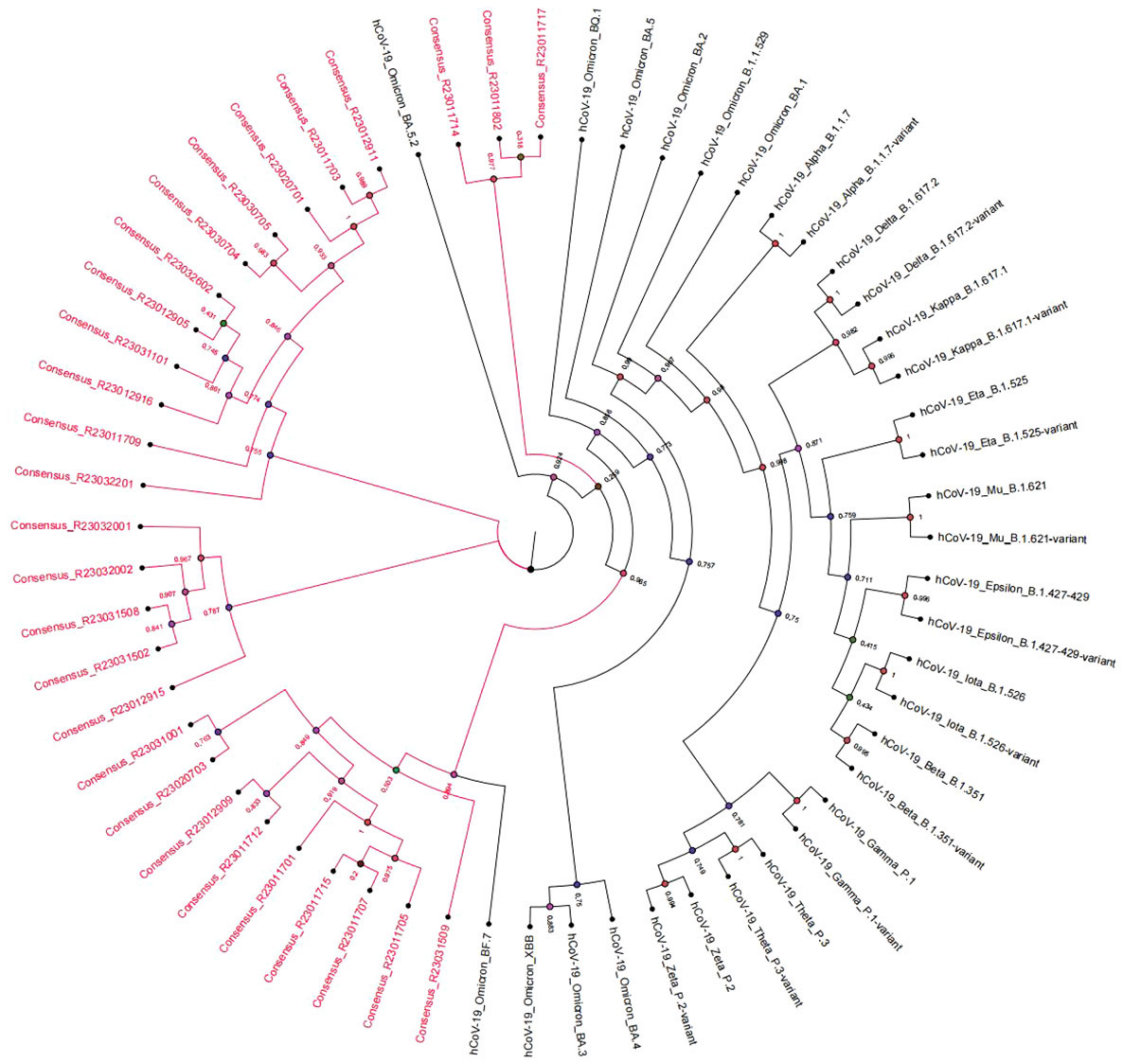
Phylogenetic tree of SARS-CoV-2 genomes representing strains and subvariants from the patients (in red).

The consensus sequences of 28 SARS-CoV-2 samples were obtained with the sequence length ranging from 29842 to 29854bp. The average sequencing depth was 32200x and the genome average coverage of sequencing was 99%. The NextClade analysis showed that the 28 SARS-CoV-2 sequences belonged to 22B (Omicron); With Pangolin typing, the subvariants were classified 5 to Omicron BA.5.2, 8 to Omicron BF.7, 1 to Omicron DY.1, 4 to Omicron DY.2, 5 to Omicron DY.3, 5 to Omicron DY.4. An evolutionary tree was built based on the complete genome sequences of COVID-19 samples. We calculated a maximum-likelihood phylogeny, including all SARS-CoV-2 genomes of interest, genomes of viruses from patients in this study were shown in red. The patients phylogenetic tree showed 3 defined clusters BF, DY and DZ (**Figure 2**). The Pangolin typing with different depth threshold (20X, 100X) showed consistent classification results.

**Figure 3:**
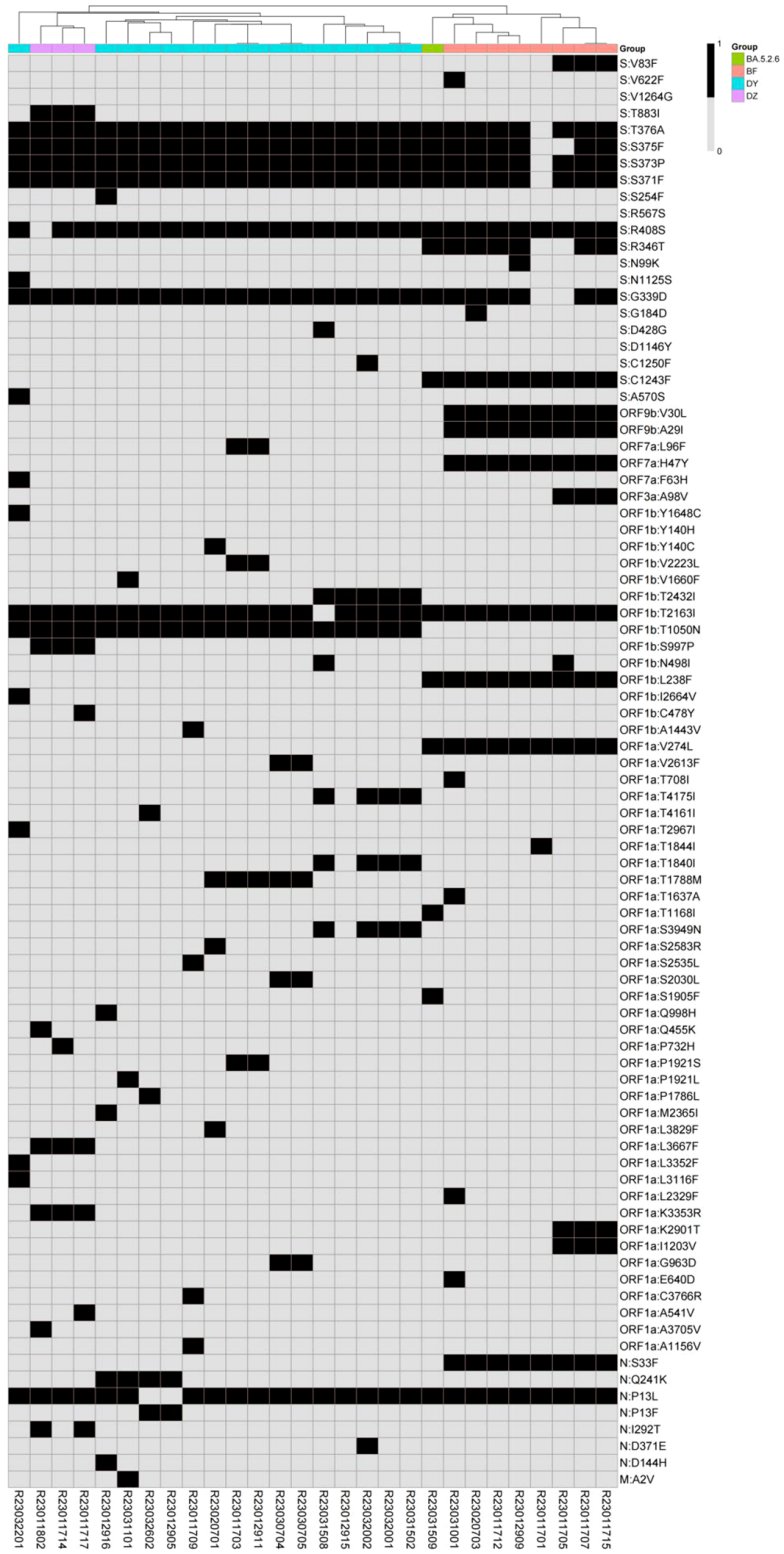
Hierarchical classification heatmap of SARS-CoV-2 specific mutations (in black) representing strains and subvariants from the patients (in color).

By comparing with the Wuhan-Hu-1 reference genome (NC_045512.2), there were 72 to 90 amino acid mutations (**Supplementary Table 2**) among the 28 SARS-CoV-2 consensus sequences. There were 43 identical amino acid mutation sites shared by all samples distributed in 8 regions: 23 in S region (Y505H, V213G, T478K, S477N, T19I, Q954H, Q498R, P681H, N969K, N764K, N679K, N501Y, N440K, L452R, K417N, H655Y, G142D, F486V, E484A, D796Y, D614G, D405N, A27S), 2 in ORF9b region (P10S, D16G), 1 in ORF3a region (T223I), 3 in ORF1b region (R1315C, P314L, I1566V), 7 in ORF1a region (T842I, T3255I, T3090I, S135R, P3395H, L3027F, G1307S), 3 in N region(S413R, R203K, G204R), 3 in M region (Q19E, D3N, A63T), and 1 in E region(T91I). A heat map was drawn for the 86 specific mutations of the 28 SARS-CoV-2 consensus sequences, the hierarchical classification based on the mutations can separate the strains and subvariants into clusters consistent to the Pangolin typing (**Figure 3**).

Referring to the mark mutation databases of the Cov-Lineages.org Lineage Report (https://cov-lineages.org/lineage_list.html), the marker mutations of the BA.5.2.48 strain were C2710T, C8626T and T17209C; the BA.5.2.49 strain were C24210T, A14673G and T16456C; the BA.5.2.6 strain was S:R346T; the DZ.1 strain was S:D1146Y; the BF.7.14 strain was S:C1243F; the BF.7.14.1 strain was S:V83F; the DY.1 strain was S:A570S; the DY.2 strain isN:Q241K; the DY.3 strain was ORF1a:T1788M; the DY.4 strain is ORF1b:T2432I. The marker mutations of 28 SARS-CoV-2 clinical samples were consistent with the mutations of each Omicron strain. Four mutations of ORF9b:V30L, ORF9b:A29I, ORF7a:H47Y and N:S33F only existed in 8 BF.7 samples; seven mutations of S:N1125S, S:A570S, ORF7a:F63H, ORF1b:Y1648C, ORF1b:I2664V, ORF1a:T2967I, ORF1a:L3352F and ORF1a:L3116F only existed in 1 DY.1 sample; one mutation of N:Q241K only existed in DY.2; 1 mutation of ORF1a:T1788M only existed in DY.3; Three mutations of ORF1b:T2432I, ORF1a:T4175I, ORF1a:T1840I and ORF1a:S3949N only existed in 6 DY.4 samples.

### 3.5 Comparison of clinical severity and lymphocyte subsets among (BA.5.2.48+BA.5.2.49+BA.5.2.6), BF.7.14 and DY subvariants

Clinical characteristics and laboratory test results of patients were analyzed in different Omicron subvariant groups, including BA.5.2.48+BA.5.2.49+BA.5.2.6 (n=5), BF.7.14 (n=8), and DY subvariant (n=15). The results showed that in all three groups patients had similar clinical manifestations, including fever, cough, expectoration, shortness of breath, headache, and fatigue, with no statistical significance between them. Most patients had pre-existing conditions such as hypertension and diabetes. Although some patients experienced complications, there was no significant difference in the distribution of severe cases among the three groups (*P>0.05*) (**Table 1**).

Patients with Omicron subvariants (BA.5.2.48+BA.5.2.49+BA.5.2.6), BF.7.14 and DY subvariants all had reduced lymphocyte counts and the levels of CRP, IL-6, PCT, DDT, and troponin were all higher than normal (**Table 2**). total lymphocyte counts, total T lymphocyte counts, NK cell counts, were all lower than normal. There was no statistical significance between the groups for most parameters (*P>0.05*) except Leukocyte and Neutrophils with P value of 0.002 and 0.009 (**Table 2**); there was also no significant difference in the PCR CT values between groups (**Table 3**).

**Figure 4:**
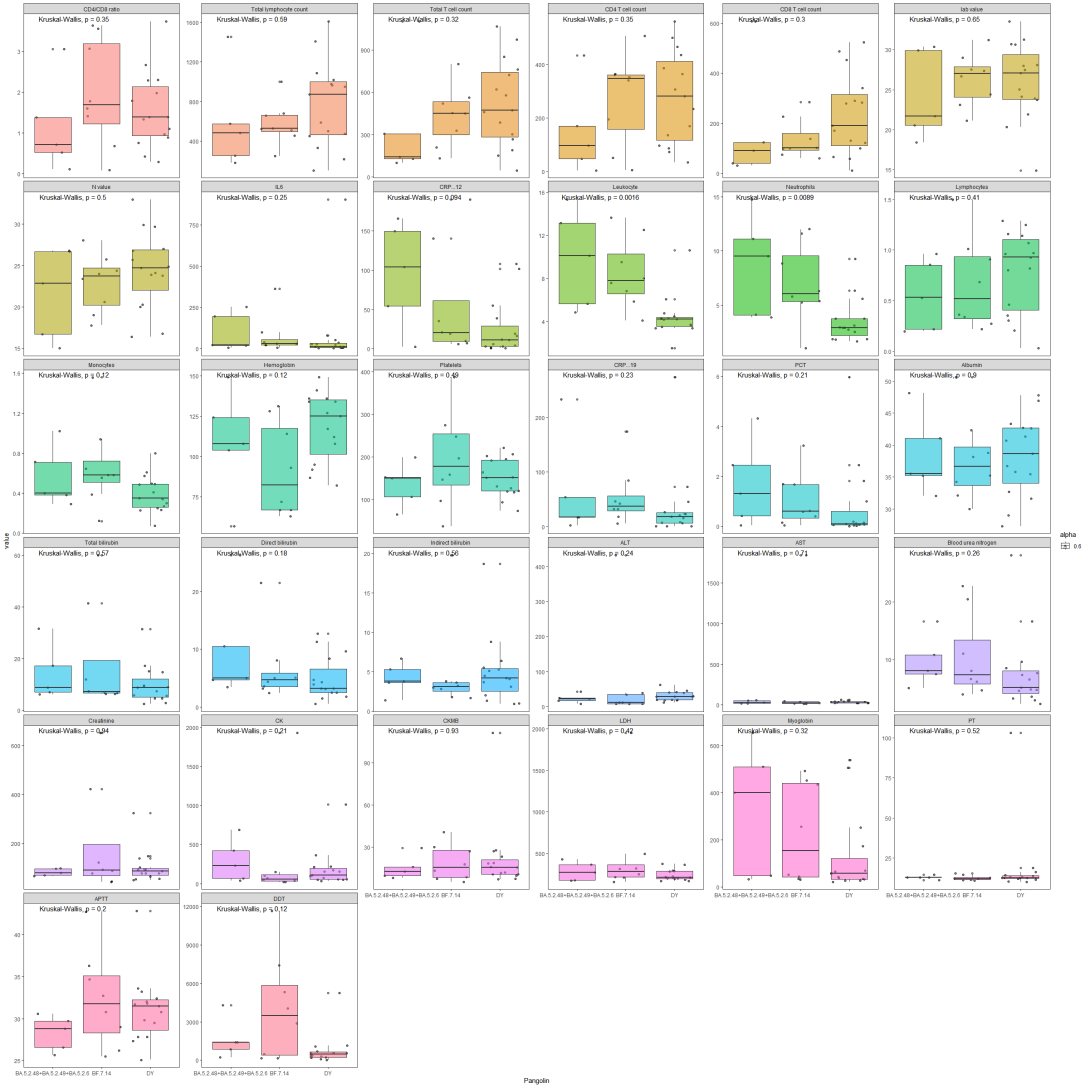
Box plots comparing the three subvariant groups for numerical laboratory testing results.

Box plots comparing the three subvariant groups for numerical laboratory testing results are shown in **Figure 4**. Wilcoxon p values are labeled for each comparison, which are consistent with the p values in **Tables 1**–**3**.

### 3.6 Correlation between mutation sites and immunological parameters

**Figure 5:**
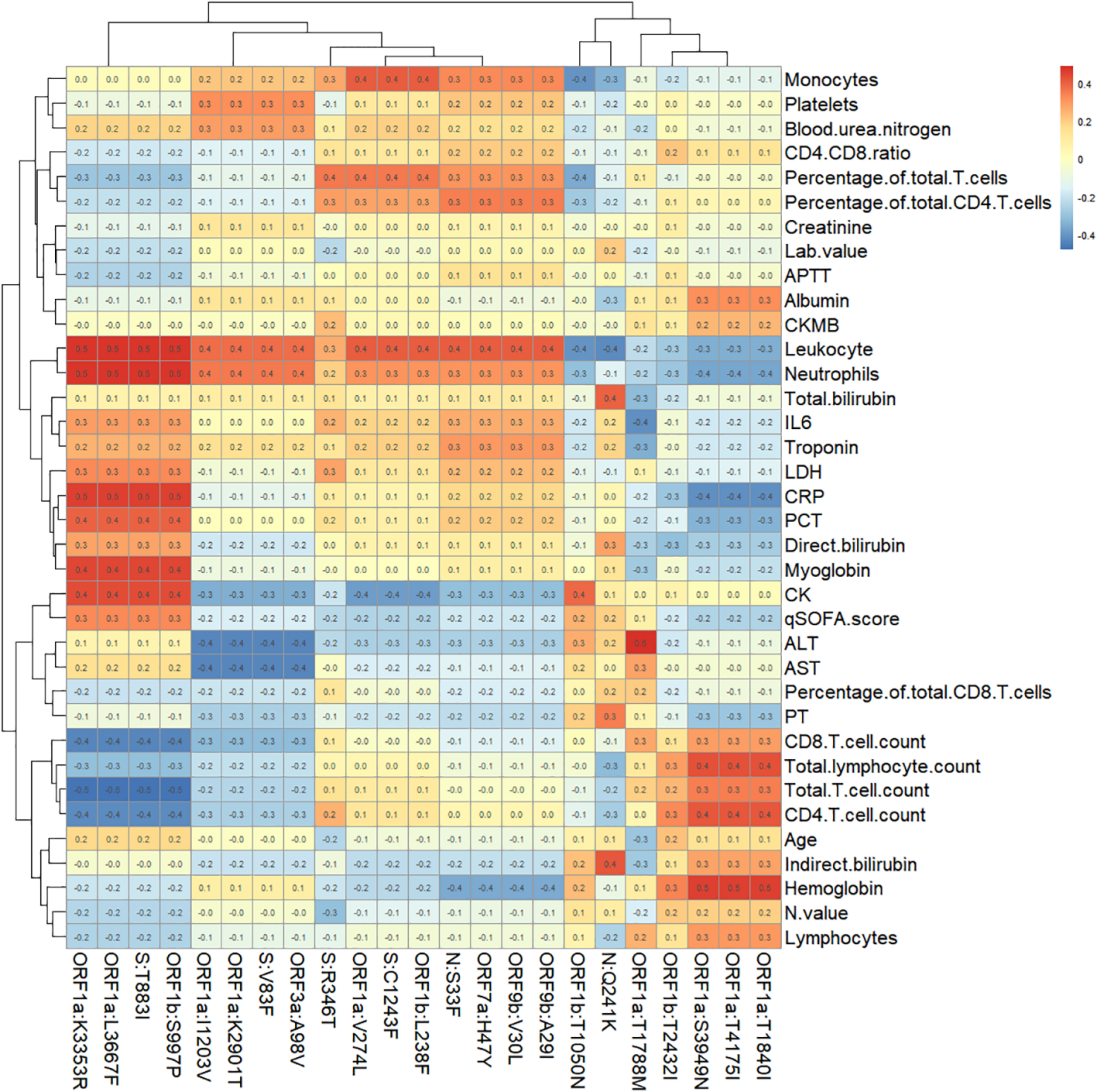
Heatmap of Spearman correlation coefficients between specific mutation sites and immunological parameters.

For the 86 specific mutations in the consensus sequences of 28 samples, we selected 23 of them which has at least 3 samples with mutation or non-mutation. Spearman Rank Correlation was performed between the mutations (1 representing mutation and 0 representing non-mutation) and the laboratory testing parameters. A heatmap of Spearman correlation coefficients between the mutations and testing parameters is displayed in **Figure 5**.

**Figure 6:**
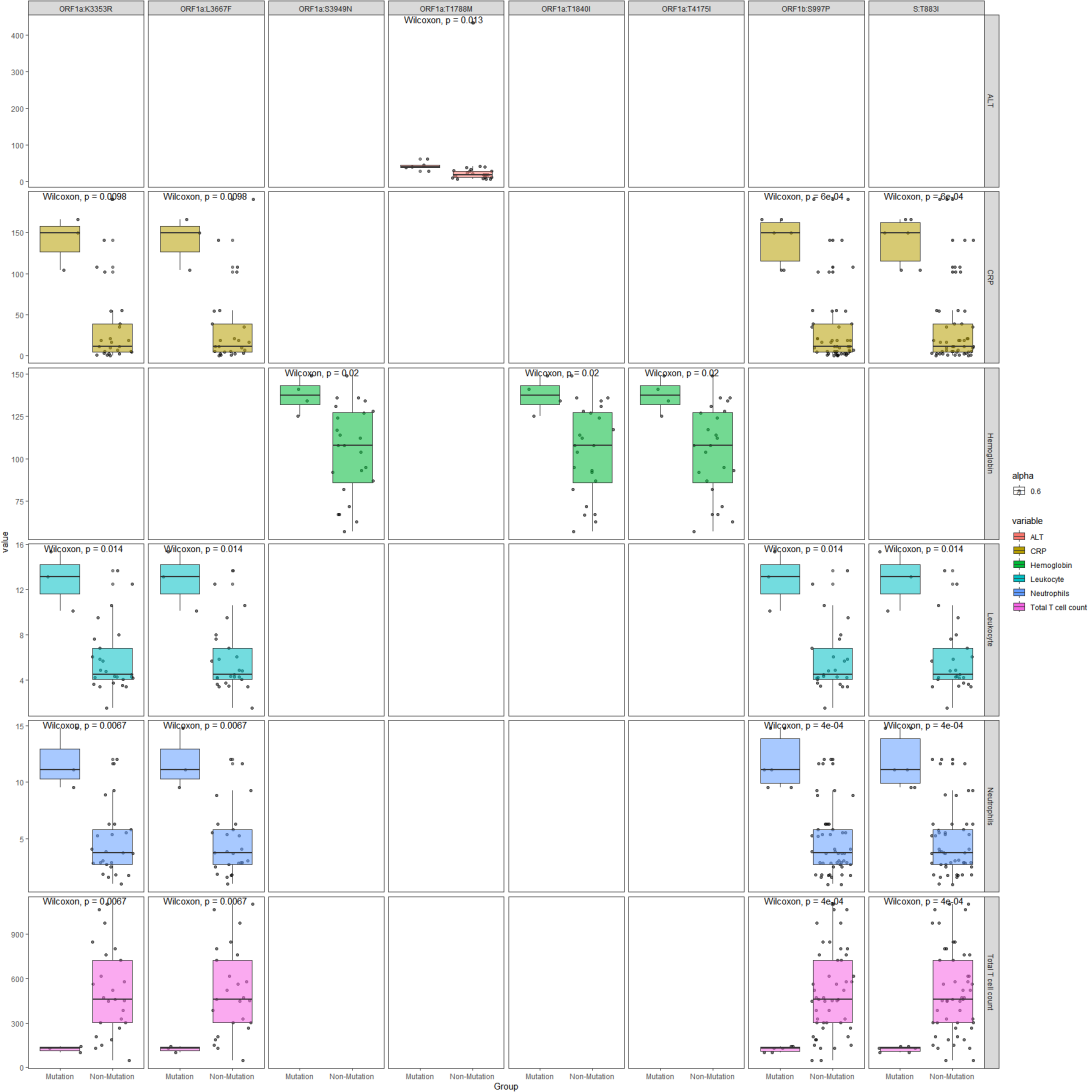
Boxplots comparing the mutation and non-mutation groups for the selected mutation sites and immunological parameters with Spearman correlation coefficient greater than 0.5.

20 pairs of mutations and testing parameters are selected with Spearman correlation coefficient greater than 0.5, which include 8 specific mutations. Box plots comparing the mutation and non-mutation groups for the selected pairs are illustrated in **Figure 6**. Wilcoxon test p values are labeled for each comparison, which showed statistical significance(P<0.05). Four mutations - ORF1a:K3353R, ORF1a:L3667F, ORF1b:S997P, S:T883I showed consistent correlation with four parameters: CRP, Leukocyte, Neutrophils, Total T cell count. Other four mutations (ORF1a:S3949N, ORF1a:T1840I, ORF1a:T4175I, ORF1a:T1788M) showed correlation with Hemoglobin or ALT only, and they only exist in DY.4 samples.

## 4 Discussion

Currently, the Omicron BA.5.2 subvariant is getting rampant in China and there are numerous studies available concerned with Omicron. However, there are no articles examining whether there are differences between clinical features, immune function, and disease severity among BA.5.2 subvariants (BA.5.2.48, BA.5.2.49, BF.7.14 and DY). Our study aims to fill this gap in knowledge.

SARS-CoV-2 attaches to the ACE2 receptor via the receptor-binding domain (RBD) on its surface, and then invades the host cells. The peripheral blood lymphocytes have been found to decrease significantly in patients with severe COVID-19 infections. Recent studies found SARS-CoV-2 potentially attacked and destroyed T lymphocytes, similar to the way human immunodeficiency virus (HIV) operates. It resulted in a decline or failure of the immune function, and in severe cases, even lead to the death of patients (Jafarzadeh et al., 2021). The severity and prognosis of the clinical condition were correlated with T cell destruction and immune response (Huang et al., 2020). However, ACE2 receptors are rarely present on the surface of T lymphocytes. It was speculated that SARS-COV-2 may bind to the CD147 receptors or integrins on the surface of T lymphocytes (Kuklina, 2022; Huang et al., 2023), leading to their invasion and subsequent destruction. This can result in a significant decrease of T lymphocyte count in the bloodstream. In present study, we found that the total lymphocyte counts, total T lymphocyte counts, CD4 cell counts, CD8 cell counts, B cell counts, and NK cell counts in patients with Omicron infection were significantly lower than normal. Compared to non-critically ill patients, critically ill patients exhibited higher levels of IL-6 and might subject to more severe cellular immunity attacks, and the level of lymphocytes decreases significantly, which was consistent with the findings of early research conducted by Liu et al (Liu et al., 2020; Liu et al., 2021). This phenomenon may be related to the hyperactivation of inflammatory factors and the excessive activation and abnormal apoptosis of lymphocytes. However, Our study found no significant difference in the lymphocyte subsets among the BA.5.2 evolutionary strains of Omicron, although all strains showed lower lymphocyte counts than normal. It may indicate that BA.5.2 evolutionary strains have roughly the same affinity and attack power toward lymphocytes, In addition, there was no significant difference in nucleic acid PCR CT values of all patients, which was consistent with the study by Shi et al (Yin et al., 2021). suggesting that their viral loads were similar, and viral load was not a reliable indicator for distinguishing disease severity and different subvariants of the Omicron.

The clinical manifestations of most patients were similar to those reported in previous studies (Huang et al., 2020). The most common symptoms on admission were fever, cough, expectoration, shortness of breath, sore throat and headache. Previous studies have identified various risk factors that were closely associated with the prognosis of patients (Shi et al., 2020; Zhou et al., 2020). These included an increase in neutrophil counts, a decrease in lymphocyte counts, higher levels of inflammatory factors such as CRP and IL-6, increased troponin, creatinine and DDT levels, as well as a higher qSOFA score. In our study, the lymphocyte counts of patients have decreased, and the levels of C-reactive protein, IL-6, procalcitonin, D-dimer, troponin, were higher than normal values. Undoubtedly, these laboratory indicators often indicate the severity of the disease, as they reflect a hyperactivation of inflammatory factors within the patient’s body, which can even result in multiple organ dysfunction. Therefore, clinicians need to be highly vigilant when the above indicators change. Furthermore, it has been observed that critically ill patients exhibit significantly higher qSOFA scores (*p<0.001*). It is important to note that the severity of organ function damage is directly proportional to the qSOFA score, indicating a higher likelihood of sepsis and septic shock. Consequently, patients with these characteristics tend to have a poorer prognosis. Previous reports have indicated that BA.5.2.48 is more likely to result in severe diseases and has a higher proportion of severe cases compared to BF.7 (Wang et al., 2023). However, our study found no significant differences in laboratory tests and qSOFA scores between different evolutionary strains of Omicron BA.5.2. Additionally, the proportion of patients with severe Omicron infection was similar across different subvariants, indicating that the disease severity and prognosis resulted by each evolutionary strain of Omicron BA.5.2 were analogical.

Mutations in the N-terminal domain (NTD) of the spike protein can alter the antigen structure of Omicron. Additionally, mutations in the RBD have been found to enhance the affinity of the virus for the human ACE2 receptors (Wang et al., 2023).The mutations can increase the infectiousness and immune escape of Omicron significantly. In this study, genomic sequences and mutation sites of viruses were identified in all patients, revealing 43 identical mutation sites across all samples, which were consistent with known mutation sites of the Omicron subvariants. We also found that specific mutations were present in different samples. The ORF9b:V30L, ORF9b:A29I, ORF7a:H47Y, and N:S33F mutations were exclusively found in the BF.7.14 sample. Similarly, S:N1125S, S:A570S, ORF7a:F63H, ORF1b:Y1648C, ORF1b:I2664V, ORF1a:T2967I, ORF1a:L3352F, and ORF1a:L3116F mutations were only present in the DY.1 samples. The N:Q241K mutation was exclusive to the DY.2 sample, while the ORF1a:T1788M mutation was found only in the DY.3 sample. And the ORF1b:T2432I, ORF1a:T4175I, ORF1a:T1840I and ORF1a:S3949N mutations were exclusively present in the DY.4 samples. We found there was no statistical significance between the three subvariant groups for most parameters (*P>0.05*) except Leukocyte and Neutrophils. Four mutations - ORF1a:K3353R, ORF1a:L3667F, ORF1b:S997P, S:T883I showed correlation with four parameters: CRP, Leukocyte, Neutrophils, Total T cell count, and there was no knowledge found about these four mutations. Another four mutations - ORF1a:S3949N, ORF1a:T1840I, ORF1a:T4175I, ORF1a:T1788M showed correlation with Hemoglobin or ALT only, and they only exist in DY.4 samples.

This study is limited by the lack of B lymphocyte percentage and count, as well as NK cell percentage and count in certain patients with the DY subvariant, meant that the immune function of these patients cannot be fully displayed. Additionally, the study has a small sample size, with only 28 patients with Omicron infection included, and it would be more persuasive if the sample was larger.

## 5 Conclusions

As far as we know, there are few articles discussing the various evolutionary branches of Omicron BA.5.2. it would assume that there were differences in clinical characteristics and disease severity among the evolutionary branches of Omicron BA.5.2, but the evidence is insufficient. Our study analyzed the genomic sequences of Omicron viruses from all enrolled patients, and explored whether there were differences in clinical characteristics, immune function, and disease severity among Omicron BA.5.2 subvariants. Four mutations - ORF1a:K3353R, ORF1a:L3667F, ORF1b:S997P, S:T883I showed correlation with immunological responses although this conclusion suffers from the small sample size. In our study, the clinical characteristics, immune function damage, and disease severity of different subvariants of Omicron BA.5.2 appeared to be similar. However, we found that critically ill patients experienced a more severe cellular immune attack and inflammatory response. In conclusion, this study presented clinical characteristics of Omicron subvariants infection in COV-19 patients and systematically analyzed important lymphocytes subsets in each clinical subgroup. It would help clinician to better management of Omicron infections in future.

## Data availability statement

The datasets presented in this study can be found in online repositories. The names of the repository/repositories and accession number(s) can be found in the article/**Supplementary Material**.

## Ethics statement

The studies involving humans were approved by Medical Ethics Committee of Zhangjiajie City People’s Hospital. The studies were conducted in accordance with the local legislation and institutional requirements. The participants provided their written informed consent to participate in this study.

## Author contributions

JH: Validation, Writing – original draft, Writing – review & editing. MX: Formal Analysis, Supervision, Validation, Writing – original draft. RL: Writing – original draft, Writing – review & editing. SW: Data curation, Methodology, Resources, Software, Supervision, Writing – original draft. XD: Data curation, Formal Analysis, Investigation, Methodology, Writing – original draft. JP: Data curation, Methodology, Supervision, Writing – original draft. EL: Conceptualization, Formal Analysis, Validation, Visualization, Writing – original draft. YZ: Conceptualization, Methodology, Supervision, Writing – original draft. CL: Methodology, Project administration, Supervision, Writing – original draft. QZ: Conceptualization, Funding acquisition, Investigation, Project administration, Supervision, Writing – review & editing. JXT: Conceptualization, Investigation, Project administration, Writing – review & editing. XW: Conceptualization, Formal Analysis, Investigation, Supervision, Writing – review & editing. ZS: Conceptualization, Data curation, Investigation, Methodology, Supervision, Writing – review & editing. JT: Conceptualization, Investigation, Supervision, Writing – review & editing. BP: Conceptualization, Data curation, Formal Analysis, Writing – original draft. JZ: Conceptualization, Investigation, Writing – original draft. JL: Conceptualization, Investigation, Writing – original draft. LD: Writing – original draft, Writing – review & editing. ML: Writing – original draft, Writing – review & editing.

## Supplementary material

Supplementary Figure 1 Patient screening and inclusion process.
