# Supplementary figures and images for: Comparative analysis of clinical and immunological profiles across Omicron BA.5.2 subvariants using next-generation sequencing in a Chinese cohort
Source: Front Cell Infect Microbiol. 2023 Oct 30;13:1288914. doi: 10.3389/fcimb.2023.1288914 (PMC10642935; doi:10.3389/fcimb.2023.1288914)

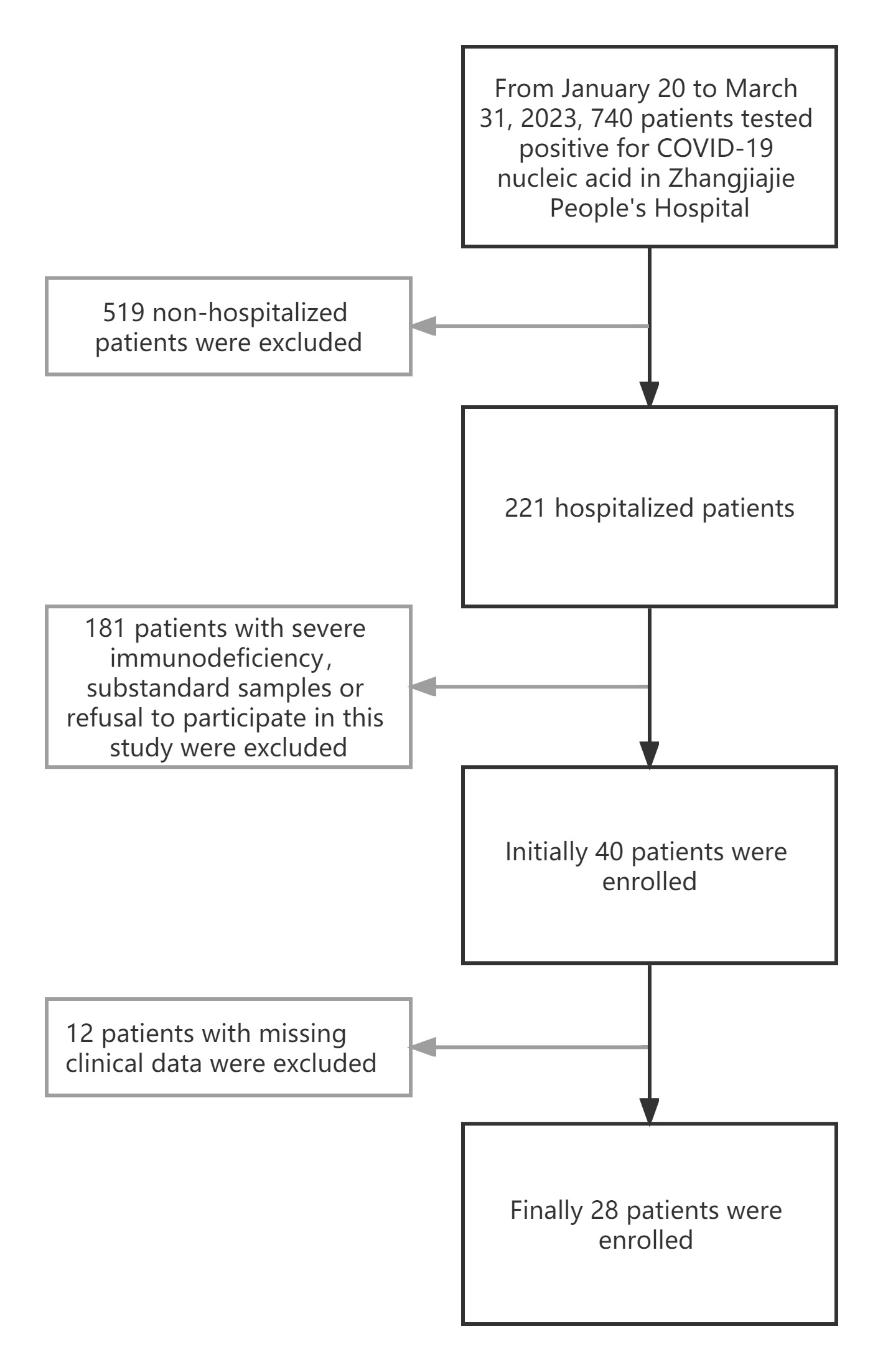

Supplement: Supplementary Figure 1 — Patient screening and inclusion process. [file Image_1.jpeg]
