# Supplementary material for: Comparative analysis of clinical and immunological profiles across Omicron BA.5.2 subvariants using next-generation sequencing in a Chinese cohort
Source: Front Cell Infect Microbiol. 2023 Oct 30;13:1288914. doi: 10.3389/fcimb.2023.1288914 (PMC10642935; doi:10.3389/fcimb.2023.1288914)
Supplement: Supplementary file 2 [file Table_1.docx]

Supplementary Table 1: Classification criteria of COVID‐19 cases

| Classification | Criteria |
| --- | --- |
| Mild | Symptomatic infection includes fever, cough, sore throat, fatigue, headache or myalgia, but there were no pneumonia signs on chest imaging. |
| Moderate | Fever and respiratory symptoms with radiological manifestations of pneumonia. |
| Severe | 1. Shortness of breath, respiratory rate (RR) ≥ 30 times/min; 2. In the resting state, when inhaling air, the oxygen saturation ≤ 93%; 3. Arterial oxygen tension (PaO_2_)/inspiratory oxygen fraction (FiO_2_) ≤ 300 mmHg; 4. Clinical symptoms progressively worsened, and lung imaging showed that the lesion had progressed significantly >50% within 24 to 48 h upon chest X-ray examination. |
| Critical | 1. Respiratory failure occurs and mechanical ventilation is required; 2. Shock occurs; 3.complicated with other organ failure that requires monitoring and treatment in Intensive Care Unit (ICU). |
