# Supplementary material for: Comparative analysis of clinical and immunological profiles across Omicron BA.5.2 subvariants using next-generation sequencing in a Chinese cohort
Source: Front Cell Infect Microbiol. 2023 Oct 30;13:1288914. doi: 10.3389/fcimb.2023.1288914 (PMC10642935; doi:10.3389/fcimb.2023.1288914)
Supplement: Supplementary file 3 [file Table_2.docx]

Supplementary Table 2: Mutations sites of 28 samples

| **Sample_name** | **Type** | **Pangolin** | **S:R346T** | **C2710T** | **C8626T** | **T17208C** | **C24210T** | **A14673G** | **T16456C** | **S:D1146Y** | **S:C1243F** | **S:V83F** | **S:A570S** | **N:Q241K** | **ORF1a:T1788M** | **ORF1b:T2432I** | **S:D1146Y** |
| --- | --- | --- | --- | --- | --- | --- | --- | --- | --- | --- | --- | --- | --- | --- | --- | --- | --- |
| R23011709 | 22B (Omicron) | BA.5.2.48 |  | √ | √ | √ |  |  |  |  |  |  |  |  |  |  |  |
| R23011714 | 22B (Omicron) | BA.5.2.49 |  |  | √ |  | √ | √ | √ |  |  |  |  |  |  |  |  |
| R23011717 | 22B (Omicron) | BA.5.2.49 |  |  | √ |  | √ | √ | √ |  |  |  |  |  |  |  |  |
| R23011802 | 22B (Omicron) | BA.5.2.49 |  |  | √ |  | √ |  | √ |  |  |  |  |  |  |  |  |
| R23031509 | 22B (Omicron) | BA.5.2.6 | √ |  |  |  |  |  |  |  | √ |  |  |  |  |  |  |
| R23011701 | 22B (Omicron) | BF.7.14 |  |  |  |  |  |  |  |  | √ |  |  |  |  |  |  |
| R23020703 | 22B (Omicron) | BF.7.14 | √ |  |  |  |  |  |  |  | √ |  |  |  |  |  |  |
| R23031001 | 22B (Omicron) | BF.7.14 | √ |  |  |  |  |  |  |  | √ |  |  |  |  |  |  |
| R23011705 | 22B (Omicron) | BF.7.14.1 |  |  |  |  |  |  |  |  | √ | √ |  |  |  |  |  |
| R23011707 | 22B (Omicron) | BF.7.14.1 | √ |  |  |  |  |  |  |  | √ | √ |  |  |  |  |  |
| R23011715 | 22B (Omicron) | BF.7.14.1 | √ |  |  |  |  |  |  |  | √ | √ |  |  |  |  |  |
| R23011712 | 22B (Omicron) | BF.7.14.5 | √ |  |  |  |  |  |  |  | √ |  |  |  |  |  |  |
| R23012909 | 22B (Omicron) | BF.7.14.5 | √ |  |  |  |  |  |  |  | √ |  |  |  |  |  |  |
| R23032201 | 22B (Omicron) | DY.1 |  | √ | √ | √ |  |  |  |  |  |  | √ |  |  |  |  |
| R23012905 | 22B (Omicron) | DY.2 |  | √ | √ | √ |  |  |  |  |  |  |  | √ |  |  |  |
| R23012916 | 22B (Omicron) | DY.2 |  | √ | √ | √ |  |  |  |  |  |  |  | √ |  |  |  |
| R23031101 | 22B (Omicron) | DY.2 |  | √ | √ | √ |  |  |  |  |  |  |  | √ |  |  |  |
| R23032602 | 22B (Omicron) | DY.2 |  | √ | √ | √ |  |  |  |  |  |  |  | √ |  |  |  |
| R23011703 | 22B (Omicron) | DY.3 |  | √ | √ | √ |  |  |  |  |  |  |  |  | √ |  |  |
| R23012911 | 22B (Omicron) | DY.3 |  | √ | √ | √ |  |  |  |  |  |  |  |  | √ |  |  |
| R23020701 | 22B (Omicron) | DY.3 |  | √ | √ | √ |  |  |  |  |  |  |  |  | √ |  |  |
| R23030704 | 22B (Omicron) | DY.3 |  | √ | √ | √ |  |  |  |  |  |  |  |  | √ |  |  |
| R23030705 | 22B (Omicron) | DY.3 |  | √ | √ | √ |  |  |  |  |  |  |  |  | √ |  |  |
| R23012915 | 22B (Omicron) | DY.4 |  | √ | √ | √ |  |  |  |  |  |  |  |  |  | √ |  |
| R23031502 | 22B (Omicron) | DY.4 |  | √ | √ | √ |  |  |  |  |  |  |  |  |  | √ |  |
| R23031508 | 22B (Omicron) | DY.4 |  | √ | √ | √ |  |  |  |  |  |  |  |  |  | √ |  |
| R23032001 | 22B (Omicron) | DY.4 |  | √ | √ | √ |  |  |  |  |  |  |  |  |  | √ |  |
| R23032002 | 22B (Omicron) | DY.4 |  | √ | √ | √ |  |  |  |  |  |  |  |  |  | √ |  |
